# Case report: Clinical, imaging, and genetic characteristics of type B niemann pick disease combined with segawa syndrome diagnosed via dual gene sequencing

**DOI:** 10.3389/fgene.2024.1391936

**Published:** 2024-05-17

**Authors:** Fang Wu, Dongying Su, Weisi Wang, Xia Song, Shufeng Fan, Jinzhan Su, Linying Ma, Jianxia Xu, Qinpan Rao

**Affiliations:** ^1^ Department of Radiology, Second Affiliated Hospital of Zhejiang University of Traditional Chinese Medicine, Hangzhou, Zhejiang, China; ^2^ Department of Respiratory, Second Affiliated Hospital of Zhejiang University of Traditional Chinese Medicine, Hangzhou, Zhejiang, China

**Keywords:** niemann pick disease, segawa syndrome, imaging manifestations, genetic features, clinical symptoms

## Abstract

Niemann Pick disease B (NPB) often presents with hepatosplenomegaly and lung pathological changes, but it usually does not present with central nervous system symptoms. This report presents the unique case of a 21-year-old woman with a 10-year history of hard skin and hepatosplenomegaly. Genetic sequencing revealed NPB and also suggested Segawa syndrome. Although symptomatic supportive treatments were administered in an attempt to improve muscle tone and treat the skin sclerosis, their efficacy was not satisfactory, and the patient refused further treatment. This case provides several noteworthy findings. First, although NPB and Segawa syndrome are rare, both are autosomal recessive inherited diseases that share common clinical symptoms and imaging manifestations. Second, when NPB and Segawa syndrome are highly suspected, screening for tyrosine hydroxylase (*TH*) and sphingomyelin phosphodiesterase-1 (*SMPD1*) gene mutations is critical to determine an accurate diagnosis. Finally, early diagnosis and comprehensive therapies are crucial for improving the prognosis of patients with NPB and Segawa syndrome.

## 1 Introduction

Niemann-Pick A/B, also known as acid sphingomyelinase deficiency (ASMD), is a rare autosomal recessive lysosomal storage caused by mutations in the *SMPD1* gene encoding the acid sphingomyelinase enzyme (Ota et al., 2020). Based on the clinical phenotype, ASMD has been divided into infantile neurovisceral Niemann-Pick A (NPA), chronic neurovisceral Niemann-Pick A/B (NPA/B), and chronic visceral Niemann-Pick B (NPB) (Geberhiwot et al., 2023). Owing to insufficient acid sphingomyelinase (ASM) activity, excessive accumulation of sphingomyelin in monocytes, macrophages, and tissues leads to clinical symptoms in NPA and NPB. NPA patients typically exhibit hepatosplenomegaly and severe central nervous system involvement in infancy, with a life expectancy rarely exceeding 2–3 years. In contrast, most patients with NPB have no evident central nervous system involvement. The disease progresses gradually, with abnormal liver function, hyperplenism, and progressive interstitial pulmonary disease, osteoporosis, and hyperlipidemia. Clinical manifestations of NPA/B fall between those of NPA and NPB, characterized by hepatosplenomegaly and slowly progressive neurological diseases ranging from mild dystonia to severe progressive neurological abnormalities. The onset of neurological symptoms in these patients is later than that in NPA and is more indolent.

Dopa-responsive dystonia (DRD), also known as Segawa syndrome, is a rare neurotransmitter disease with a prevalence rate of 0.5 per one million people (Dobricic et al., 2017) The associated decrease in dopamine caused by a mutation in the gene encoding tyrosine hydroxylase (*TH*) may lead to dystonia, tremor, and severe encephalopathy (Wang et al., 2022)This report presents the case of a patient with concurrent NPB and Segawa syndrome. The 21-year-old female presented with a 10-year history of facial skin tightness, gradual hardening of her skin, disappearance of her forehead lines, deformation of her finger joints in both hands, and shortness of breath, with no apparent cause. She did not experience any other discomfort, such as cold, fever, cough, phlegm, or stomach or wrist pain, and had not undergone treatment for any of her symptoms.

## 2 Case description

Two weeks prior to her presentation, the patient sought outpatient treatment. At that time, she had an elevated erythrocyte sedimentation rate (42 mm/h) and immunoglobulin G levels (21.30 g/L). The patient also presented with positive anti-nuclear antibodies, weakly positive antiribonucleoprotein, and weakly positive anti-Smith antibody. The patient was believed to have scleroderma, and she was admitted to the hospital with skin arthralgia and connective tissue disease. The patient denied any relative family history and reported that her parents and sister were in good health. Physical examination revealed a saddle nose, facial skin tightening and hardening, the disappearance of forehead wrinkles, a swan neck-like deformity of the fingers on both hands, and raised arches of both feet.

**FIGURE 1:**
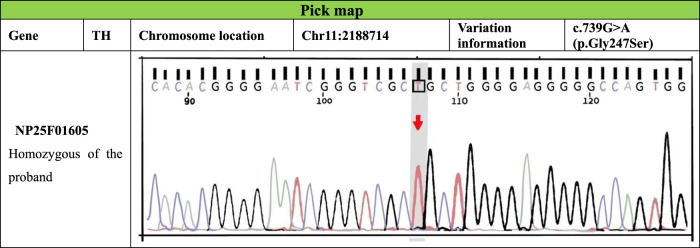
Electrophoretic map of *TH* gene mutation in patients.

**FIGURE 2:**
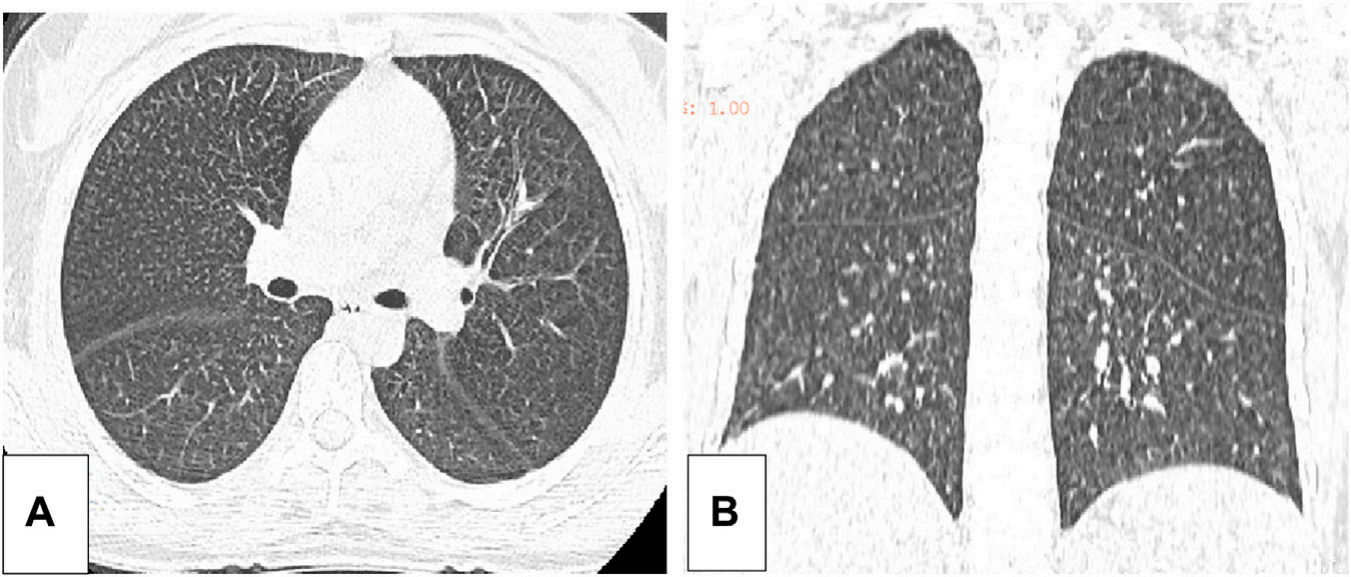
**(A)** The axial position of the high resolution thoracic CT of the patient. **(B)** The coronal plane of the high resolution CT of the patient.

**FIGURE 3:**
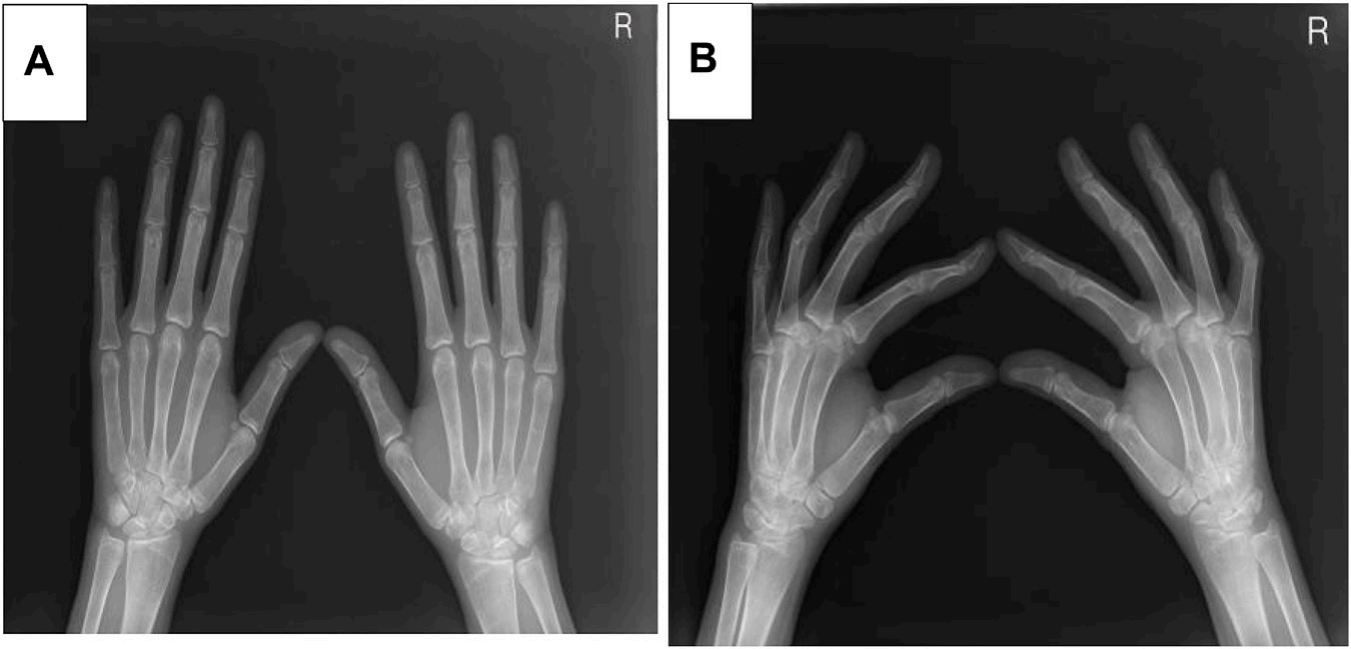
**(A)** The X-ray positive position of the patient. **(B)** The X-ray lateral position of the patient.

**FIGURE 4:**
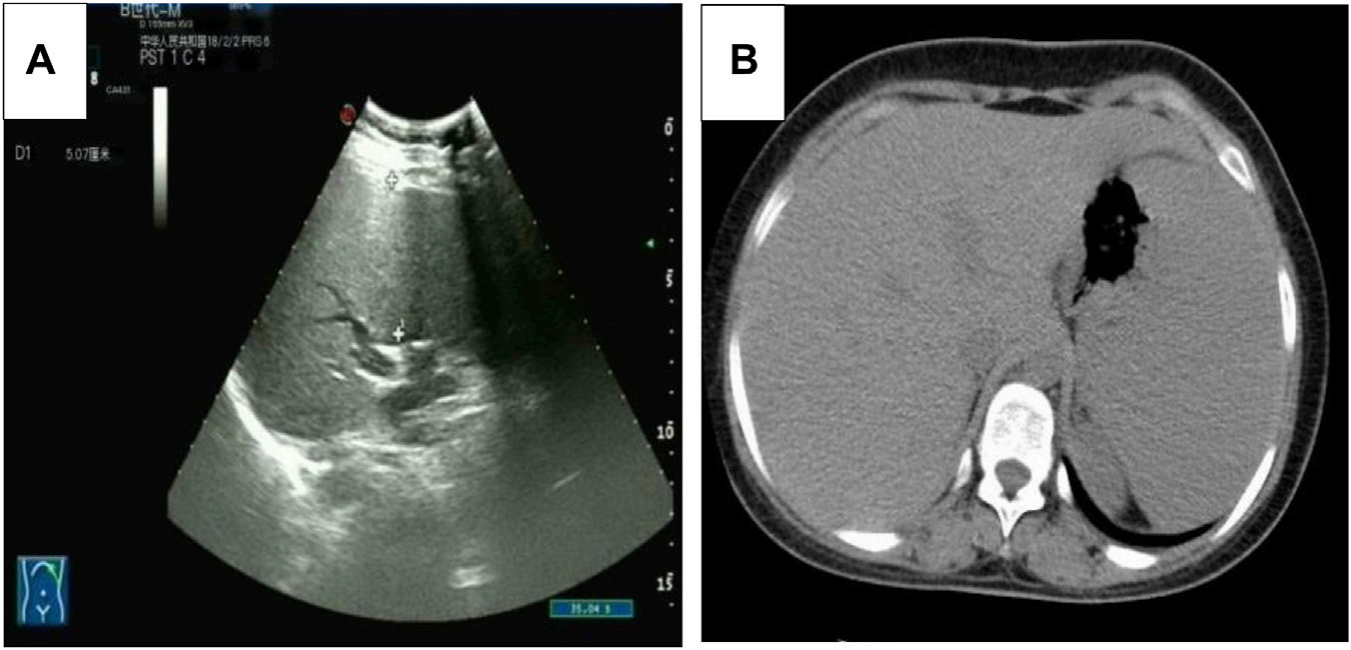
**(A)** The abdominal ultrasound image of the patient. **(B)** The axial abdominal CT image of the patient.

Five months after her presentation, whole exome sequencing revealed a variant in the *SMPD1*gene (chromosomal position chr11: 6415105_6415108, variant information NM: 000,543.5: c.1341-21_1341-18del, genetic pattern AR, homozygote, ACMG variant classification pathogenic variation) and Segawa syndrome [variant gene *TH*, chromosomal position chr11: 2188714, variant information NM_199,292.3: c.739G>A (p.Gly247Ser), genetic pattern AR, homozygote, ACMG variant classification pathogenic variation] (Figure 1). High-resolution chest computed tomography (CT) revealed diffuse miliary nodules distributed along the bronchial vascular tree in both lungs, with regular lung morphology and relatively uniform density. The interlobular septum exhibited slight local thickening (Figure 2). Plain radiography of the patient’s hands revealed that the bone trabeculae of both hands were slightly sparse, density was slightly reduced, the cortex was thinner, the marrow cavity was widened, and the alignment of the distal interphalangeal joints of the left index and middle fingers and the proximal interphalangeal joints of the right little finger showed swan neck-like changes (Figure 3). Abdominal ultrasonography and CT revealed enlargement of the liver and spleen (Figure 4).

The patient was diagnosed with ASMD 8 months after her presentation. She exhibited decreased plasma acid sphingomyelinase activity (0.18 μmol/L/h, normal value: 1.19–13.97 μmol/L/h), haemoglobin levels (97 g/L, normal value:115–150 g/L), white blood cell count (2.4 × 10^9^/L, normal value:3.5-9.5 × 10^9^/L) and platelet count (86 ×10^9^/L, normal value:125-350 × 10^9^/L). Conversely, the activities of alanine aminotransferase (ALT) (67 U/L, normal value:7–40 U/L) and aspartate aminotransferase (AST) (64 U/L, normal value:13–35 U/L) were elevated.

After a comprehensive evaluation and discussion of the clinical, imaging, and genetic data, the patient was diagnosed with NPB combined with Segawa syndrome. The patient was administered L-dopamine in an attempt to improve her muscle tone disorders. The initial dose of L-dopamine was 0.25 g, once every 12 h, which was taken after meals. The follow-up dose was increased every 3–7 days according to the patient’s symptoms, ranging from 0.125 to 0.75 g per day, with a maximum of 6 g per day, until the ideal curative effect was achieved. However, the patient chose to discontinue treatment after 15 days as the therapeutic effects were not satisfactory.

## 3 Discussion

Low ASM activity is characteristic of NPA and NPB. Quantification of ASM activity in convenient cells, such as circulating white blood cells or cultured skin fibroblasts (He et al., 2003), has been used as a diagnostic test for NPA and NPB. Sequencing of *SMPD1* can also be used to confirm NPA and NPB (Schuchman et al., 2016). The *SMPD1* pathogenic variant c. 1,341-21_1341-18del was previously reported for a case of NPB in which the patient exhibited decreased ASM activity, normal intelligence, no central nervous system symptoms, and negative imaging findings (Lidove et al., 2015). The presentation of the current patient was consistent with NPB. The ASM activity in patients with NPA/B is very broad (Mihaylova et al., 2007).

Additional manifestations of low ASM activity in patients with ASMD include bone and joint pain, bone mass reduction, and foam cells that can be easily detected in the bone marrow. In the current patient, hand radiography revealed sparse bone trabeculae, reduced bone density, which are known bone manifestations of ASMD (Simpson et al., 2010). These manifestations are believed to be caused by foam macrophages infiltrating the bone marrow. A previous study reported joint and bone pain and an increased incidence of fractures in patients with NPB (Wasserstein et al., 2013). However, the current patient did not have bone or joint pain.

Because of the cellular abnormalities in the liver and spleen, patients with ASMD with low ASM activity exhibit liver and spleen enlargement and haematological and lipid abnormalities (McGovern et al., 2004). The current patient had liver and spleen enlargement and elevated AST and ALT levels, indicating abnormal liver function. Thrombocytopenia is another common finding, and some patients may have extremely low high-density lipoprotein cholesterol and high triglyceride levels. The patient in this report had a low platelet count, although her cholesterol and triglyceride levels were normal.

In patients with ASMD, sphingolipids are often deposited in the pulmonary macrophages with diffuse infiltration in the lungs, resulting in an increased risk of infection. The current patient reported shortness of breath after exercise. A pulmonary function examination indicated mild mixed-ventilation dysfunction, and CT analysis revealed local thickening of the pulmonary interlobular septa and miliary nodules, which were similar to other recently reported lung imaging results (Ufuk, 2024). Our findings were considered to be caused by foamy macrophage infiltration. No specific therapy for this infiltration is available worldwide. Bone marrow transplantation and lung transplantation have been reported in patients with ASMD; however, these treatments do not cure the disease (Victor et al., 2003; Mora et al., 2022). Enzyme replacement therapy (ERT) reduces sphingomyelin accumulation in the lungs, liver, spleen, and other non-central nervous system (CNS) organs and clinical trials have largely proved its efficacy on non-CNS manifestations. Indeed, it is not suitable for treating ASMD CNS involvement since the enzyme do not cross the blood brain barrier (Geberhiwot et al., 2023). ERT has been approved by the Food and Drug Administration (FAD), and the European Medical Agency (EMA).

The patient in this report was also diagnosed with Segawa syndrome, which is primarily caused by reduced dopamine synthesis. Clinical manifestations include gradually worsening postural tremors, gait abnormalities, and lower limb muscle tone disorders in early childhood or adolescence. Patients also experience limb stiffness, slow movement, and loss of facial expression. However, the patient’s language skills and intelligence are not affected. A previous study (Yang et al., 2018) reported that the incidence of Segawa syndrome is higher in women than in men and that the clinical symptoms are more severe in women. Laboratory examinations are typically normal in patients with Segawa syndrome. The patient in this report developed tight and hard facial skin, the disappearance of her forehead lines, stiffness of her limbs, swan neck-like deformities of the fingers on both hands, and elevated arches of both feet. Genetic testing revealed a mutation in *TH*. Most types of Segawa syndrome are associated with autosomal dominant mutations in the gene encoding GTP cyclohydrolase-1 (*GCH1*), and rarely by recessive mutations in *TH*. Low TH activity leads to sustained damage of catecholaminergic neurotransmitters, prenatal brain developmental disorders, and postpartum growth disorders (Kuwabara et al., 2018)This may result in muscle tone disorders, tremors, and severe encephalopathy. L-dopamine is an effective drug for the treatment of Segawa syndrome; however, the treatment effect in this case was not acceptable and the patient was transferred to another hospital. The subsequent treatment plan was not made clear, but the patient has returned to everyday life and work.

With the development of genetic testing technology and an improved understanding of genetic diseases, the presence of more than one rare genetic disease in the same patient has been reported (Cianci et al., 2020; Saettini et al., 2020; Tan et al., 2023). Over 5% of patients who underwent whole-exome sequencing had a bimolecular diagnosis or multipoint genomic variation (Posey et al., 2017). When two rare genetic diseases occur together, the clinical phenotype is complex, and few guidelines exist for the diagnosis and treatment ofdiagnosing and treating concurrent genetic diseases. When the patient’s clinical manifestations and disease progression are not completely consistent with the diagnosis ofdiagnosing a rare genetic disease, the possibility of a second rare genetic disease should be considered. In addition, while bimolecular diagnosis impacts the clinical treatment of patients, it can also be used in proactive intervention in the early stage of the disease, with potential targeting or therapeutic effects, and. It may provide a risk estimate of recurrence.

This study has several limitations. First, owing to the lack of computer analysis tools, we could not carry out a functional prediction of the pathogenic mutations in *SMPD1* and *TH*; thus, the potential functional value of the two related diseases is not discussed. Second, the patient was only tested for ASM activity and not that of TH; thus, the activity of TH was not addressed in this report.

In summary, to achieve an accurate diagnosis with curately diagnose both ASMD and Segawa syndrome, the clinical manifestations, imaging findings, laboratory examination, and molecular genetic analyses must be understood.

## 4 Patient perspective

The patient provided informed consent for the publication of this report.

## Data Availability

The data presented in the study are deposited in the figshare, Doi: 10.6084/m9.figshare.25795561, https://figshare.com/articles/journal_contribution/Case_report_Clinical_imaging_and_genetic_characteristics_of_type_B_niemann_pick_disease_combined_with_segawa_syndrome_diagnosed_via_dual_gene_sequencing/25795561?file=46238359.

## References

[B1] Cianci P. Pezzoli L. Maitz S. Agosti M. Iascone M. Selicorni A. (2020). Dual genetic diag noses: neurofibromatosis type 1 and kbg syndrome. Clin. Dysmorphol. 29 (2), 101–103. 10.1097/MCD.0000000000000296 31567426

[B2] Dobricic V. Tomic A. Brankovic V. Kresojevic N. Jankovic M. Westenberger A. (2017). Gch1 mutations are common in Serbian patients with dystonia-parkinsonism: challenging previously reported prevalence rates of dopa-responsive dystonia. Park. Relat. Disord. 45, 81–84. 10.1016/j.parkreldis.2017.09.017 28958832

[B3] Geberhiwot T. Wasserstein M. Wanninayake S. Bolton S. C. Dardis A. Lehman A. (2023). Consen-sus clinical management guidelines for acid sphingomyelinase deficiency (niemann-pick disease types a, b and a/b). Orphanet J. Rare Dis. 18 (1), 85. 10.1186/s13023-023-02686-6 37069638 PMC10108815

[B4] He X. Chen F. Dagan A. Gatt S. Schuchman E. H. (2003). A fluorescence-based, high-performance liquid chromatographic assay to determine acid sphingomyelinase activity and diagnose types a and b niemann-pick disease. Anal. Biochem. 314 (1), 116–120. 10.1016/s0003-2697(02)00629-2 12633609

[B5] Kuwabara K. Kawarai T. Ishida Y. Miyamoto R. Oki R. Orlacchio A. (2018). A novel compound heterozygous th mutation in a Japanese case of dopa-responsive dystonia with mild clinical course. Park. Relat. Disord. 46, 87–89. 10.1016/j.parkreldis.2017.10.019 29126763

[B6] Lidove O. Sedel F. Charlotte F. Froissart R. Vanier M. T. (2015). Cirrhosis and liver failure: ex panding phenotype of acid sphingomyelinase-deficient niemann-pick disease in adulthood. JIMD Rep. 15, 117–121. 10.1007/8904_2014_306 24718843 PMC4270874

[B7] McGovern M. M. Pohl-Worgall T. Deckelbaum R. J. Simpson W. Mendelson D. Desnick R. J. (2004). Lipid abnormalities in children with types a and b niemann pick disease. J. Pediatr. 145 (1), 77–81. 10.1016/j.jpeds.2004.02.048 15238911

[B8] Mihaylova V. Hantke J. Sinigerska I. Cherninkova S. Raicheva M. Bouwer S. (2007). Highly variable neural involvement in sphingomyelinase-deficient niemann-pick disease caused by an ancestral gypsy mutation. Brain 130, 1050–1061. Pt 4. 10.1093/brain/awm026 17360762

[B9] Ota S. Noguchi A. Kondo D. Nakajima Y. Ito T. Arai H. (2020). An early-onset neu ronopathic form of acid sphingomyelinase deficiency: a smpd1 p.c133y mutation in the saposin domain of acid sphingomyelinase. Tohoku J. Exp. Med. 250 (1), 5–11. 10.1620/tjem.250.5 31941852

[B10] Posey J. E. Harel T. Liu P. Rosenfeld J. A. James R. A. Coban A. Z. (2017). Resolution of disease phenotypes resulting from multilocus genomic variation. N. Engl. J. Med. 376 (1), 21–31. 10.1056/NEJMoa1516767 27959697 PMC5335876

[B11] Saettini F. L'Imperio V. Fazio G. Cazzaniga G. Mazza C. Moroni I. (2020). More than an 'atypical' phenotype: dual molecular diagnosis of autoimmune lymphoproliferative syndrome and becker muscular dystrophy. Br. J. Haematol. 191 (2), 291–294. 10.1111/bjh.16967 33460031

[B12] Schuchman E. H. Wasserstein M. P. (2016). Types A and B niemann-pick disease. Pediatr. Endocrinol. Rev. PER. 13 (Suppl 1), 674–681. 10.1016/j.beem.2014.10.002 27491215

[B13] Simpson W. J. Mendelson D. Wasserstein M. P. McGovern M. M. (2010). Imaging manifestations of niemann-pick disease type b. AJR Am. J. Roentgenol. 194 (1), W12–W19. 10.2214/AJR.09.2871 20028884

[B14] Tan D. D. Liu Y. D. Fan Y. B. Wei C. J. Song D. Y. Yang H. P. (2023). Clinical and genetic characteristics of 9 rare cases with coexistence of dual genetic diagnoses. Zhonghua Er Ke Za Zhi 61 (4), 345–350. 10.3760/cma.j.cn112140-20220922-00827 37011981

[B15] Ufuk F. (2024). Pulmonary involvement of niemann-pick disease. Radiology 310 (2), e232633. Case Reports; Journal Article. 10.1148/radiol.232633 38349239

[B16] Villemagne V. L. Velakoulis D. Dore V. Bozinoski S. Masters C. L. Rowe C. C. (2019). Imaging of tau deposits in adults with niemann-pick type c disease: a case-control study. Eur. J. Nucl. Med. Mol. Imaging. 46 (5), 1132–1138. 10.1007/s00259-019-4273-7 30690666

[B17] Wang Y. Wang C. Liu M. Xu W. Wang S. Yuan F. (2022). Segawa syndrome caused by th gene mutation and its mechanism. Front. Genet. 13, 1004307. 10.3389/fgene.2022.1004307 36568392 PMC9772685

[B18] Wasserstein M. Godbold J. McGovern M. M. (2013). Skeletal manifestations in pediatric and adult patients with niemann pick disease type b. J. Inherit. Metab. Dis. 36 (1), 123–127. 10.1007/s10545-012-9503-0 22718274

